# Association of retinopathy severity with cardiovascular and renal outcomes in patients with type 1 diabetes: a multi-state modeling analysis

**DOI:** 10.1038/s41598-022-08166-4

**Published:** 2022-03-09

**Authors:** Wei-Ming Wang, Huang-Tz Ou, Miin-Jye Wen, Pei-Fang Su, Chen-Yi Yang, Te-Hui Kuo, Ming-Cheng Wang, Wei-Hung Lin

**Affiliations:** 1 Department of Statistics and Institute of Data Science, College of Management, National Cheng Kung University, Tainan, Taiwan; 2 Institute of Clinical Pharmacy and Pharmaceutical Sciences, College of Medicine, National Cheng Kung University, Tainan, Taiwan; 3 Department of Pharmacy, College of Medicine, National Cheng Kung University, Tainan, Taiwan; 4 Department of Pharmacy, National Cheng Kung University Hospital, Tainan, Taiwan; 5 Institute of International Management, College of Management, National Cheng Kung University, Tainan, Taiwan; 6 Department and Institute of Public Health, College of Medicine, National Cheng Kung University, Tainan, Taiwan; 7 Department of Internal Medicine, College of Medicine, National Cheng Kung University Hospital, National Cheng Kung University, 138 Sheng-Li Road, Tainan, 70428 Taiwan; 8 Institute of Clinical Medicine, College of Medicine, National Cheng Kung University, Tainan, Taiwan

**Keywords:** Endocrinology, Nephrology, Risk factors

## Abstract

This study aimed to assess the impact of diabetic retinopathy (DR) severity on the incidence of major adverse cardiac events (MACE) and end-stage renal disease (ESRD) in T1D patients. Patients diagnosed with T1D between 1999 and 2013 were identified from patient-level data of Taiwan’s National Health Insurance Research database. A total of 1135 patients were included and classified into mild DR (n = 454), severe DR (n = 227), or non-DR (n = 454) by using propensity score matching. Multi-state model analyses, an extension of competing risk models with adjustment for transition-specific covariates for prediction of subsequent MACE and ESRD, were performed. MACE and ESRD risks were significantly higher in the severe DR patients; a 2.97-fold (1.73, 5.07) and 12.29-fold (6.50, 23.23) increase in the MACE risk among the severe DR patients compared to the mild DR and DR-free patients, respectively; and, a 5.91-fold (3.50, 9.99) and 82.31-fold (29.07, 233.04) greater ESRD risk of severe DR patients than that of the mild DR and DR-free groups, respectively (*p* < 0.001). Severity of DR was significantly associated with the late diabetes-related vascular events (i.e., MACE, ESRD) among T1D patients.

## Introduction

Type 1 diabetes (T1D) is associated with a higher age-adjusted mortality compared to the general population^1,2^. A nationwide study conducted in Taiwan has reported a significant increase in the annual number of patients with T1D diagnosed between 1999 and 2010, raising awareness regarding T1D-related morbidities and mortalities^3^. People diagnosed with T1D before the age of 30 years have been estimated to have a 4.7-fold excess mortality risk^4^. And, the patients with early diagnosis of T1D (i.e., 0–12 years old) have better life expectancy and lower lifetime costs compared to those with the late diagnosis (i.e., after 12 years old)^5^.

T1D-related complications typically occur in both the macro- (e.g., coronary artery disease [CAD]) and micro-vasculatures (e.g., proliferative diabetic retinopathy [PDR], diabetic nephropathy [DN]). Among them, CAD and DN are the major causes of morbidity and mortality in patients with T1D^6^. Hyperglycemia is a leading pathogenic factor of micro-angiopathic disease^7^, while diabetic retinopathy (DR) is one of the leading microvascular complications among T1D patients^8^. It is evident that approximately 50% of people with T1D becomes advanced DR after 40 years of diabetes^9^. DR progression starts as mild non-proliferative abnormalities, and evolves into moderate and severe non-proliferative DR with vascular closure and eventually PDR^10^. DN is a leading cause of end-stage renal disease (ESRD) and seems to occur as a result of an interaction among inflammatory, metabolic, and hemodynamic factors^11^. Previous epidemiologic reports have confirmed that the presence of DR was correlated with risk of cardiovascular events^12,13^ and contributes to an excess risk of heart failure among diabetic patients^14^.

Long-term estimates of PDR and vascular events among patients with T1D are scare due to the lack of systematic long-term follow-up data for these patients. Taiwan has a very high prevalence of ESRD, with diabetes being a leading cause^15^. Large-scale population-based studies with long-term follow-up are therefore warranted to expand our understanding of the role of T1D in ESRD in ethnic Chinese populations. Using a large population-based T1D cohort in Taiwan, we have previously reported the long-term risk of ESRD and associated mortality among T1D patients across all ages^3,16^ and details of the cumulative incidences of various vascular complications in this population^17^. In this study, we further applied advanced multi-state model analyses to explore the impact of DR severity on long-term risks of ESRD and major adverse cardiovascular events (MACE) among patients with T1D by utilizing a nation-wide population-based T1D patient cohort.

## Methods

The Institutional Review Board of National Cheng Kung University Hospital approved the study before commencement (B-EX-103-015). All study analyses were conducted based on retrospective data with de-identified patient-level records, so informed consents to individual patients were exempted by the Research Ethics Committees from the Institutional Review Board of National Cheng Kung University Hospital. Also, all methods in this study were performed in accordance with the relevant guidelines and regulations.

### Data sources

Taiwan’s National Health Insurance (NHI) program was launched since 1995. The National Health Insurance Research Database (NHIRD), a large-scale claims-based database supervised by the Bureau of NHI, Department of Health, and maintained by the Institutes of NHI, is provided to scientists in Taiwan for research purposes. The NHIRD includes all inpatient and ambulatory medical claims for about 99% of the Taiwanese population. Information in the NHIRD has been confirmed to be accurate and complete^18,19^.

### Identification of study patients with newly diagnosed T1D

We identified T1D cases diagnosed during 1999 to 2013 in the NHIRD using the International Classification of Diseases, 9^th^ Revision, Clinical Modification, ICD-9-CM disease code = 250. In Taiwan, the certifications of various catastrophic illnesses (e.g., T1D, ESRD) are subject to critical evaluations and reviews by the Bureau of NHI. Because patients receiving catastrophic illness certificates (CICs) are eligible for exemption from insurance premiums and co-payments, the data associated CIC records are highly accurate and reliable^3^. We thus considered the cases with the first registered CICs for T1D as newly diagnosed T1D patients after 1999 so that those with the registered CICs as T1D before 1999 were excluded. Patients with T1D were the patients who had three or more outpatient T1D diagnoses with insulin prescriptions and a history of diabetic ketoacidosis, a positive glucagon test, or the presence of GAD antibodies^20^; these procedures have been previously validated using medical chart reviews with a positive predictive value of 98.3%^3^.

### Definition of DR

DR was defined as the presence of typical retinal microvascular lesions, including hemorrhages, microaneurysms, exudates, and fibrous proliferation, laser marks, a previous history of vitreous hemorrhage, and retinal surgery in an individual with diabetes. DR was measured according to ICD-9-CM disease codes 362.01-362.07. In our study, severe retinopathy was further measured based on the status of laser photocoagulation treatment as previous study^21^; i.e., patients with severe DR were defined as those receiving laser treatment at three months before or after DR diagnosis for severe non-PDR, PDR, or diabetic maculopathy. Patients with mild DR were those with background DR and preproliferative DR^21^.

### Definitions of MACE and ESRD

The primary endpoint was the occurrence of a MACE, defined as a composite of myocardial infarction, congestive heart failure, peripheral vascular disease, and cerebrovascular disease (ICD-9-CM codes: 410, 412 for myocardial infarction; 402.01, 402.11, 402.91, 425, 428, 429.3 for congestive heart failure; 440, 441, 442, 443.1–443.9x, 447.1x, 785.4x for peripheral vascular disease; 362.34, 430–436, 437–437.1, 437.9, 438, 781.4, 784.3, 997.0 for cerebrovascular disease) requiring the first hospitalization or two outpatient visits within 1 year. In addition, those who had MACE-related diagnoses in their inpatient or outpatient records before enrollment were excluded because of potential confounding. ESRD was another outcome of interest, which was defined using the CIC registration records with the ICD-9-CM disease code of 585. The CIC records are valid and reliable data sources for T1D and ESRD retrieval^3,22^. Since we were interested in the incident event of ESRD after T1D diagnosis, only the first registered CICs for ESRD after T1D diagnosis was considered in this study.

### Matching

Considering that the duration of diabetes possibly influences the onset and progression of DR, we first matched individual severe DR cases with mild DR or DR-free cases at a ratio of 1:2 based on diabetes duration with an interval of ± 1 month. In addition, because patients with different severities of DR are likely to vary by clinical characteristics and comorbidities, we applied propensity score matching to achieve a greater level of baseline comparability between patients with different DR severities. Here, the propensity score was defined as the probability of being severe DR versus mild DR (or non-DR) for a patient based on the given clinical characteristics and comorbidities, which was estimated using logistic regression analysis. The comorbidities in the present study were defined based on the disease categories according to Charlson’s comorbidity index (CCI) where individual comorbidity was classified as yes or no in the analysis. The comorbidities measured in this study included diabetes, hypertension, ischemic heart disease, dyslipidemia, myocardial infarction, heart failure, peripheral artery disease, stroke, dementia, chronic lung disease, peptic ulcer disease, liver disease, hemiplegia/paraplegia, and malignancy from one year before T1D diagnosis. The selection of study subjects and matching process are presented in Supplementary Figure 1.

### Statistical analysis

Baseline descriptive data are presented as the means ± standard deviations for continuous variables, and as numbers and percentages for categorical variables. The clinical characteristics of the groups were compared using the chi-square test or Fisher exact test for categorical variables and the t-test for continuous variables.

**Figure 1:**
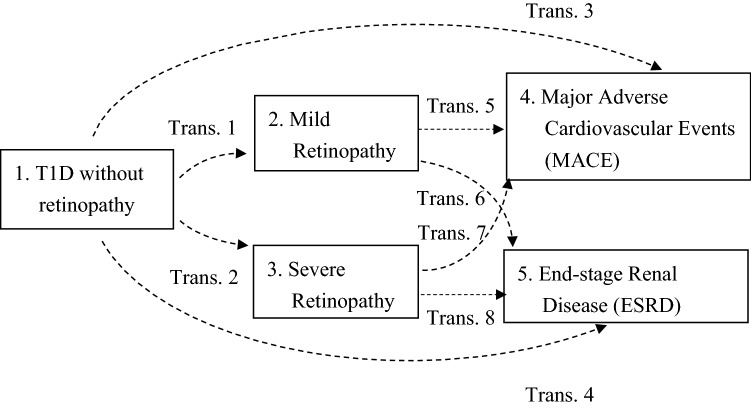
Five-state model with 8 transition for type 1 diabetes patients. All patients started in State 1. Trans 1: patients moved to mild DR form type 1 DM; Trans 2: patients moved to severe DR form type 1 DM; Trans 3: patients moved to MACE form type 1 DM; Trans 4: patients moved to ESRD form type 1 DM; Trans 5: patients moved to MACE form mild DR; Trans 6: patients moved to ESRD form mild DR; Trans 7: patients moved to MACE form severe DR; Trans 8: patients moved to ESRD form severe DR.

In the present study, patients were followed from registration of T1D to the occurrence of ESRD or MACE. In our longitudinal medical records, patients were observed over time and covariate information was collected at several occasions. Therefore, to obtain more biological insights into the disease process of a patient, we applied a multi-state model analysis with two intermediate events as mild and severe DR. A multi-state model forms an extension to that of competing risk models, where the model captures the risks that might be affected by several intermediate effects on study outcomes simultaneously^23,24^. The multi-state model is for time-to-event data in which all individuals start in one starting state and end up in several mutually exclusive absorbing states (end states), while some individuals may be censored before they reach an absorbing state. Different from traditional competing risk models which only consider one starting state and absorbing state without any intermediate events, the multi-state model can take account of the impact from intermediate effects on absorbing states (i.e., study outcomes) concurrently. Applying this modeling analysis is appropriate when the disease course or progression to be modelled includes an intermediate event of interest. In the present study, we considered the following five-states model where all patients were assumed to start from State 1 (see Fig. 1):

- State 1. T1D without retinopathy (starting state);
- State 2. Occurrence of mild DR (intermediate event);
- State 3. Occurrence of severe DR (intermediate event);
- State 4. Occurrence of MACE (absorbing/final state);
- State 5. Occurrence of ESRD (absorbing/final state).

Our multi-state model therefore consisted of 8 transitions between these states. We first considered the transition probability using Markov models, in which we ignored the influence of covariates, to estimate the transition probability from one state to another. The Markov property assumes that the probability of being a future state depends only on the present state (i.e., not the history)^24^. Further, we used the probabilities from Markov models to estimate the cause-specific transition hazards by the Nelson-Aalen estimator statistics^25^. In our data, this cause-specific cumulative hazard quantity describes the cumulative monthly risk of moving from one state to another. Next, in order to model the effect of covariates on the cause-specific hazards, a transition-specific Cox model analysis is performed in the multi-state model. Such a model specifies different covariate effects for different transitions in the absorbing states with or without intermediate events. In our data, we tested for age and gender as covariates separately to determine whether their effects on all disease transitions are consistent using a likelihood ratio test.

Moreover, the traditional Cox proportional hazard model was performed with adjustment for the same covariates (i.e., sex and gender) specified in the transition-specific Cox model abovementioned. Results from traditional and transition-specific Cox models were presented as hazard ratios (HRs) and 95% confidence intervals (CIs), and compared to examine if the impact attributed by the intermediate effects on the disease progression (which were captured in the transition-specific models) exists.

Statistical analyses in this study were conducted using SAS 9.3 statistical software (SAS Institute Inc., Cary, NC) and the mstate package in *R* 3.6.0 software, which is available from the *R* homepage (http://cran.r-project.org).

## Results

Table 1 shows the clinical characteristics of T1D patients (including 454 with mild DR, 227 with severe DR, and 454 with non-DR) after the matching. Severe DR patients were significantly older than those with mild or no DR. There were significantly fewer male patients with severe DR compared to those with no DR. There were no significant differences in comorbidities, CCI, and diabetes duration between the severe DR, mild DR, and DR-free groups after the matching.

**Table 1:** Type 1 Diabetes patients divided into mild, severe, no retinopathy groups according to propensity score matching.

Variable	Severe retinopathy (n = 227)	Mild retinopathy (n = 454)	No retinopathy (n = 454)
Age at type 1 diabetes diagnosis, years (mean ± SD)	26.6 ± 11.0	24.2 ± 11.9*	21.5 ± 11.1^†^
< 15	31 (13.2)	128 (28.2)	169 (37.2)
15–29	114 (50.2)	192 (42.3)	186 (41.0)
29–44	65 (28.6)	105 (23.1)	88 (19.4)
45 +	17 (7.5)	29 (6.4)	11 (2.4)
Male	88 (38.8)	186 (41.0)	221 (48.7)^†^
Comorbidity			
Dementia	0 (0.0)	1 (0.2)	0 (0.0)
Chronic pulmonary disease	11 (4.9)	22 (4.9)	16 (3.5)
Rheumatologic disease	2 (0.9)	2 (0.4)	3 (0.7)
Peptic ulcer disease	15 (6.6)	22 (4.9)	29 (6.4)
Mild liver disease	2 (0.9)	6 (1.3)	5 (1.1)
Hemiplegia or paraplegia	1 (0.4)	1 (0.2)	1 (0.2)
Renal disease	2 (0.9)	6 (1.3)	6 (1.3)
Malignancy	2 (0.9)	5 (1.1)	5 (1.1)
Moderate or severe liver disease	0 (0.0)	0 (0.0)	0 (0.0)
Metastatic solid tumor	0 (0.0)	0 (0.0)	0 (0.0)
AIDS	0 (0.0)	0 (0.0)	0 (0.0)
Charlson’s comorbidity index	3.18 ± 0.51	3.17 ± 0.50	3.17 ± 0.50
3	198 (87.2)	396 (87.2)	396 (87.2)
4	20 (8.8)	43 (9.5)	44 (9.7)
> 4	9 (4.0)	15 (3.3)	14 (3.1)
Diabetes duration, months	64.8 ± 45.4	64.5 ± 44.9	64.8 ± 45.3
Follow-up period, month (median (IQR))	128.7 (92.0, 155.8)	119.6 (81.1, 145.8)	68.5 (33.5, 120.1)

SD, standard deviation; IQR, interquartile range.

Diabetes duration: from T1DM diagnosed until enrol in the study.

Follow-up period: from T1DM diagnosed to MACE or ESRD.

*p < 0.05, comparison between severe retinopathy and mild retinopathy.

^†^*p* < 0.05, comparison between severe retinopathy and no retinopathy.

**Figure 2:**
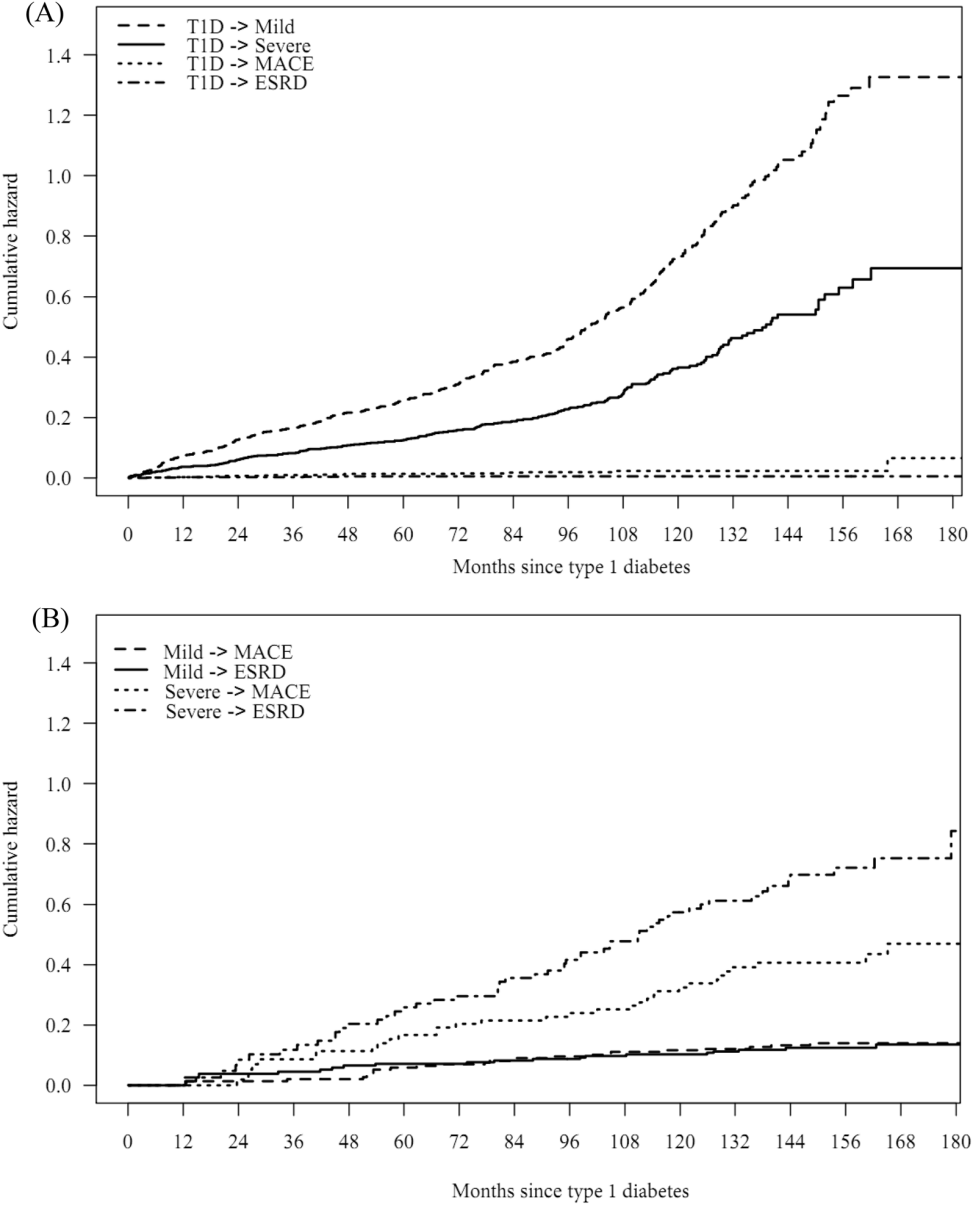
(**A**) Cumulative hazard of transition from type 1 diabetes (T1D) to mild diabetic retinopathy (DR), severe DR, end-stage renal disease (ESRD) or major adverse cardiac event (MACE). (**B**) Cumulative hazard of transition from mild DR or severe DR to ESRD or MACE.

Figure 2 shows the estimated cumulative hazards at all event times. It shows how the prognosis of a patient depends on the starting state and the moment taken as the starting point for prediction. According to the results in Fig. 2A, the transition hazard from T1D to retinopathy is higher than those to MACE or ESRD. Furthermore, Fig. 2B shows that a patient had a higher transition hazard from T1D through severe DR to MACE or ESRD than from T1D through mild DR to MACE or ESRD.

**Figure 3:**
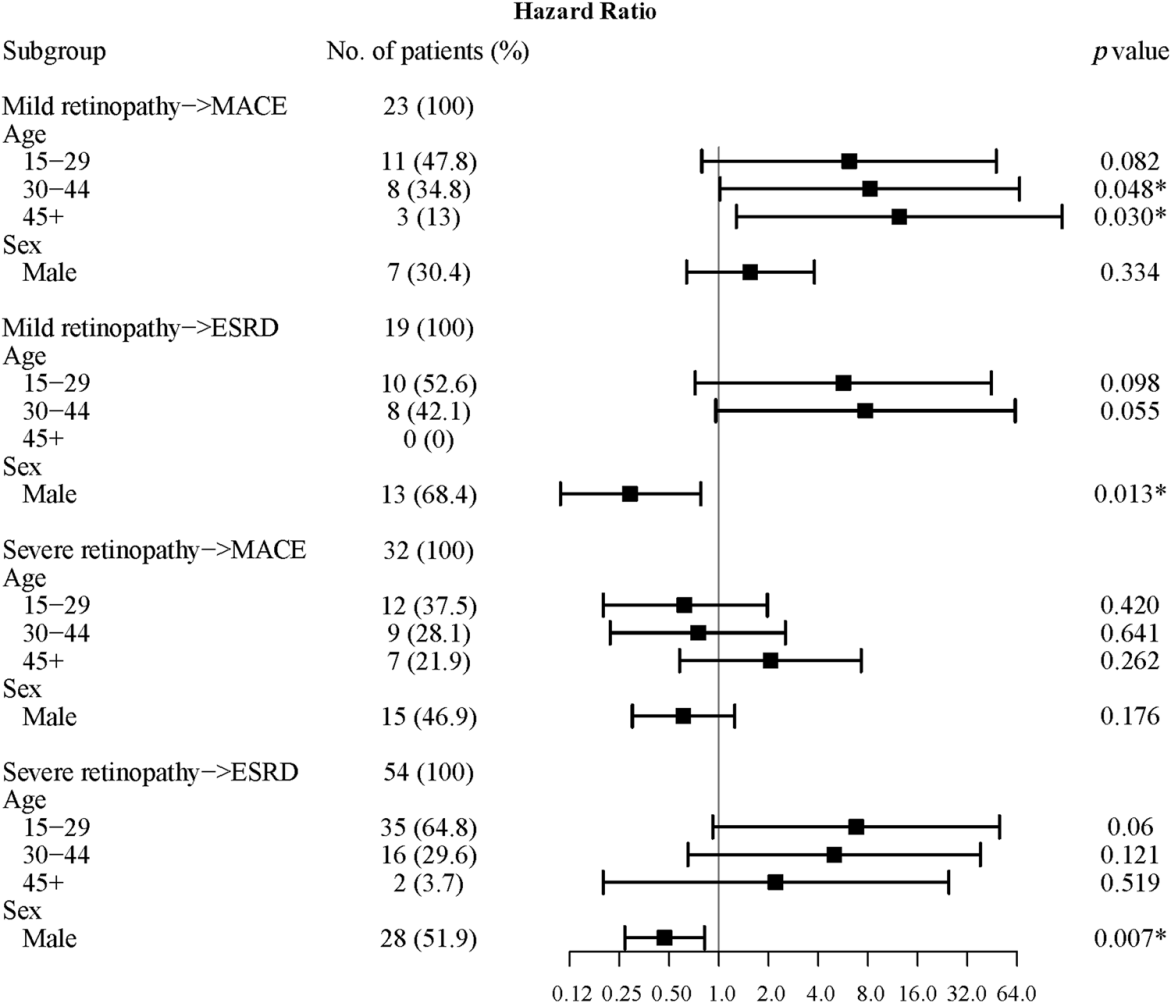
Forest plot of transition hazard between mild retinopathy, severe retinopathy, end-stage renal disease (ESRD), and major adverse cardiac event (MACE), stratified by age at type 1 diabetes diagnosis and sex.

Next, we took transition-specific covariates into account using the multi-state model analyses. Figure 3 using a forest plot displays the estimated effects of the covariates for all the transitions. We observed a significantly higher risk for MACE form mild DR as getting older above 30 years old, but not significantly for ESRD. And, the males had significantly lower risk for ESRD regardless of being from mild DR or severe DR.

Table 2 shows the results based on multi-state model analyses. The adjusted HRs of MACE and ESRD were significantly higher among the severe DR group; a 2.97-fold (1.73, 5.07) and 12.29-fold (6.50, 23.23) increase in the MACE risk in the severe DR patients compared to that of the mild DR and DR-free patients, respectively (*p* < 0.001); and a 5.91-fold (3.50, 9.99) and 82.31-fold (29.07, 233.04) greater ESRD risk of severe DR patients than that of the mild DR and DR-free groups, respectively (*p* < 0.001). Consistent results with multi-state model analyses were shown according to traditional Cox model analyses (Supplementary Table 1) but the magnitude of the risks was lesser in the traditional analyses. The adjusted HRs of MACE and ESRD were significantly higher among the severe DR group; a 2.05-fold (1.12, 4.96) and 2.91-fold (1.70, 4.98) increase in the MACE risk in the severe DR patients compared to that of the mild DR and DR-free patients, respectively (*p* < 0.001); and, a 5.21-fold (3.46, 9.53) and 17.24-fold (6.13, 47.62) greater ESRD risk of severe DR patients than that of the mild DR and DR-free groups, respectively (*p* < 0.001).

**Table 2:** Hazard ratios of end-stage renal disease (ESRD) and major adverse cardiac events (MACE) through severe and mild retinopathy from multi-state model.

Outcome: MACE	Adjusted HR (95% CI)	*p* value
Severe retinopathy (ref. = mild retinopathy)	2.97 (1.73, 5.07)	< 0.001
Severe retinopathy (ref. = no retinopathy)	12.29 (6.50, 23.23)	< 0.001

Outcome: ESRD	Adjusted HR (95% CI)	*p* value
Severe retinopathy (ref. = mild retinopathy)	5.91 (3.50, 9.99)	< 0.001
Severe retinopathy (ref. = no retinopathy)	82.31 (29.07, 233.04)	< 0.001

Adjusted hazard ratios were estimated from multi-state models adjusted for age at type 1 diabetes diagnosis and sex.

## Discussion

To our knowledge, this study is the first to examine the effect of DR severity on MACE and ESRD in a T1D patient population with applying advanced multi-state model analyses to gain more biological insights in the disease process of a patient (i.e., how a certain prognostic factor [mild or severe DR] influences the different phases of disease progression). The results show that the severity of DR was a significant risk factor for MACE and ESRD in T1D patients. Compared with mild DR, the presence of severe DR was associated with about a three-fold increase in the MACE risk and a six-fold greater ESRD risk, independent of demographic and clinical factors. These indicate that DR severity may provide additional information regarding the development of severe cardiovascular and microvascular events among patients with T1D.

The clinical manifestation and severity of DR generally vary among diabetic patients, and therefore, the different types or severities of DR should be regarded as transitions during disease progression. Conventionally, the Cox proportional hazards model analyses are generally applied to analyze the risks of severe diabetes-related complications (e.g., MACE, ESRD) that may be attributable to different DR severities. However, traditional Cox proportional hazards model analyses generally do not consider the information on intermediate events during the disease progression. Often, intermediate events change with the natural course of disease progression so that the role of some of the prognostic factors may not be the same after that event occurs. For example, having DR among patients with severe DR (e.g., PDR) may be more strongly related to future development of MACE compared to that in mild DR cases (e.g., NPDR). Therefore, we applied advanced multi-state model analyses to explore how DR severity as an intermediate state affects the risks of development of severe micro- and macro-vascular complications. Multi-state models, an extension of competing risk models, provide a framework that allows for analyzing survival data where different intermediate events can occur during the disease progression^23^. We further found that the traditional Cox model analyses yielded lower magnitudes of MACE and ESRD risks than that of the multi-state model analyses. This may be explained by the fact that compared with the traditional method, the multi-state model analyses can account for the covariate effects (i.e., age, gender) changing over time and yield the estimated effects of the covariates simultaneously for all disease transitions, but a traditional Cox model has no these considerations. In our study, we found significantly fewer male patients with severe DR and the males had significantly lower risk for ESRD regardless of being from mild DR or severe DR. Previous studies have found that women diagnosed as diabetes have a higher risk of developing chronic kidney disease later in life. Although the underlying etiology is not well known, reductions in effective renal blood flow and increases in renal vascular resistance and filtration fraction were found in female adolescents with T1D during clamped hyperglycemia^26,27^. Thus, a longer follow-up study will be needed to clarify the gender differences in the prevalence of DR and ESRD. However, it is worthy for more aggressive risk factor intervention of women with T1D to avoid the afterwards advanced microvascular complications.

DR is a common diabetes-related microvascular complication and is a leading cause of blindness in people of working ages in industrialized countries^28^. The presence of DR reflects the dysfunction of coronary microcirculation to some extent. The mechanisms underlying this relationship are not established but possible etiologies include microvascular injury-associated with aging, hypertension, atherosclerosis, and other vascular changes that may exist in the retina and other vascular beds such as the heart, brain, and kidneys^29^. Although PDR fails to independently predicting fatal and nonfatal cardiovascular diseases among T1D patients in the EURODIAB^30^, this study confirms a significant association of DR severity with MACE in an Asian patient population with T1D. Close follow-ups and timely interventions for patients with DR in clinical practice should be considered.

DR is probably the most accurate single predictor for DN; however, this relationship is not previously established yet among T1D patients. Reports from the Action to Control Cardiovascular Risk in Diabetes (ACCORD) study in patients with early DN and the Reduction of Endpoints in NIDDM with the Angiotensin II Antagonist Losartin (RENAAL) study in patients with advanced DN both found that DR was associated with composite renal endpoints^31,32^. A recent study of patients with advanced chronic kidney diseases from Taiwan also supported an association between DR and ESRD^33^. These findings suggest a potential common pathway between DR and renal diseases; DR may be representative of systemic microvascular damage secondary to diabetes, leading to both the progression of renal dysfunction and the breakdown of the blood vessel–retinal tissue barrier^34^. We therefore analyzed the prediction of DR severity for ESRD among T1D patients. DR severity may be accelerated by poor glycemic and hypertension control, which have been identified as risk factors for DR progression^35^. Early detection of DR could remind clinical physicians to be aware of future risks of developing cardiovascular events and ESRD, and thus early intervene with effective treatments (e.g., ACE inhibitors) can be given to prevent these severe complications^36,37^.

The strengths of our study include its nationwide population-based cohort design, relative long duration of follow-up (up to 12 years), and large number of incident cases of T1D according to sex and age stratifications. Also, the registration system of catastrophic diseases in Taiwan was applied to ensure the validity of disease diagnoses (i.e., T1D, ESRD). Moreover, the multi-state model analyses offered several advantages against more commonly used methods in survival analyses. Compared to the conventional Cox models, this advanced analysis is more flexible and comprehensive that can account for the effects of different baseline hazards on disease transitions and allow all sequences of events to be analyzed in a single model. One major advantage of a multi-state model compared with standard methods is that it yields the estimates of probabilities of being in all disease states (i.e., intermediate and absorbing states). In the present study, the intermediate events (mild and severe DR) in the multi-state model provide detailed information for the cardiovascular and ESRD process. And, when analyzing the risks of different intermediate events, the estimates are also adjusted with consideration of competing risk. Additionally, we implemented the information from time-varying covariates in the multi-state model analyses to ensure precision in predicting disease progression.

However, there are some study limitations to be considered. First, the claims data do not provide detailed laboratory results (e.g., HbA1c), which made it difficult to determine the severity of diabetes and comprehensively measure the predictors for MACE and ESRD. Second, no information on the true underlying cause of ESRD is available in the NHI claims; therefore, we were unable to estimate the cause-specific risks of MACE and ESRD in T1D patients. Third, T1D is a rare disease with the prevalence about 0.5% among all diabetes in Taiwan. And, the rates of study events in this population are relatively low, which may lead to large variances in study estimates.

In conclusion, the multi-state model analyses offer flexibility for studying the effects of covariates of interest on various transition rates. These models also allow to consider more biological insights about the impact of intermediate disease states on disease progression, which however are typically ignored using a traditional approach. Therefore, advanced multi-state model analyses may be alternative approaches to supplement additional information to traditional model analyses (e.g., Cox models). Moreover, considering higher MACE and ESRD risks associated with severe DR, early detection of DR for timely interventions and treatments is of importance to prevent the development of future severe diabetes vascular complications and associated fatal deaths.

## Supplementary Information

Supplementary Information.
