# Supplementary material for: Association of retinopathy severity with cardiovascular and renal outcomes in patients with type 1 diabetes: a multi-state modeling analysis
Source: Sci Rep. 2022 Mar 9;12:4177. doi: 10.1038/s41598-022-08166-4 (PMC8907198; doi:10.1038/s41598-022-08166-4)
Supplement: Supplementary file 1 — Supplementary Information. [file 41598_2022_8166_MOESM1_ESM.docx]

**SUPPLEMENTARY MATERIALS**

Supplementary Table 1:

Hazard ratios of end-stage renal disease (ESRD) and major adverse cardiac events (MACE) through severe and mild retinopathy from Cox proportional hazards model.

| Outcome: MACE | Adjusted HR (95% CI) | *p* value | |
| --- | --- | --- | --- |
| Severe retinopathy  (ref. = mild retinopathy) | 2.05 (1.12, 4.96) | 0.020 | |
| Severe retinopathy  (ref. = no retinopathy) | 2.91 (1.70, 4.98) | <0.001 | |
| Outcome: ESRD | Adjusted HR (95% CI) | | *p* value |
| Severe retinopathy  (ref. = mild retinopathy) | 5.21 (3.46, 9.53) | | <0.001 |
| Severe retinopathy  (ref. = no retinopathy) | 17.24 (6.13, 47.62) | | <0.001 |

Adjusted hazard ratios were estimated from Cox proportional hazards model models adjusted for age at type 1 diabetes diagnosis and sex.

Medical claims data from Taiwan’s National Health Insurance Research Database (NHIRD) between 1995 and 2013

N=2,254,119

Prevalent type 1 diabetes (T1D) (confirmed by Catastrophic Illness Certificate) between 1995 and 2013

N=11,643

Newly diagnosed type 1 diabetes (T1D) between 1999 and 2013

N=8,788

Exclusion of patients with any of the following criteria:

- With end stage renal disease before the index date (n=20)
- With major adverse cardiovascular events before the index date (n=399)
- With diabetic retinopathy (DR) before the index date (n=560)
- With laser treatment before the index date (n=2)

Study population N=7,807 before matching

- Severe DR (N=263)
- Mild DR (N=1,264)
- DR-free (N=6,280)

1:1:2 matching

Study population N=908

- Severe DR (N=227)
- Mild DR (N=227)
- DR-free (N=454)

Supplementary Figure 1: Flow diagram of study subject selection process
